# Supplementary material for: Pyruvate Dehydrogenase Kinase 1 inhibition mediated oxidative phosphorylation enhancement in cartilage promotes osteoarthritis progression
Source: BMC Musculoskelet Disord. 2023 Jul 20;24:597. doi: 10.1186/s12891-023-06585-6 (PMC10357736; doi:10.1186/s12891-023-06585-6)
Supplement: Supplementary file 1 — Additional file 1. [file 12891_2023_6585_MOESM1_ESM.zip › MF.Gsea.1653627316242/GOMF_METHYLATED_HISTONE_BINDING.html]

Details for gene set GOMF\_METHYLATED\_HISTONE\_BINDING[GSEA]

|  || Dataset | 55457\_collapsed\_to\_symbols.cls.cls #OA\_versus\_normal.cls.cls #OA\_versus\_normal\_repos |
| Phenotype | cls.cls#OA\_versus\_normal\_repos |
| Upregulated in class | normal |
| GeneSet | GOMF\_METHYLATED\_HISTONE\_BINDING |
| Enrichment Score (ES) | -0.40667385 |
| Normalized Enrichment Score (NES) | -1.6145095 |
| Nominal p-value | 0.007836991 |
| FDR q-value | 0.1436999 |
| FWER p-Value | 0.99 |
Table: GSEA Results Summary

  

Fig 1: Enrichment plot: GOMF\_METHYLATED\_HISTONE\_BINDING      
 Profile of the Running ES Score & Positions of GeneSet Members on the Rank Ordered List

  

| SYMBOL | TITLE | RANK IN GENE LIST | RANK METRIC SCORE | RUNNING ES | CORE ENRICHMENT || 1 | KAT8 | lysine acetyltransferase 8 [Source:HGNC Symbol;Acc:HGNC:17933] | 595 | 0.625 | -0.0138 | No |
| 2 | ING4 | inhibitor of growth family member 4 [Source:HGNC Symbol;Acc:HGNC:19423] | 680 | 0.598 | 0.0107 | No |
| 3 | DPF2 | double PHD fingers 2 [Source:HGNC Symbol;Acc:HGNC:9964] | 854 | 0.554 | 0.0261 | No |
| 4 | CBX1 | chromobox 1 [Source:HGNC Symbol;Acc:HGNC:1551] | 1331 | 0.453 | 0.0125 | No |
| 5 | CBX4 | chromobox 4 [Source:HGNC Symbol;Acc:HGNC:1554] | 1397 | 0.443 | 0.0305 | No |
| 6 | RRP8 | ribosomal RNA processing 8 [Source:HGNC Symbol;Acc:HGNC:29030] | 1673 | 0.402 | 0.0300 | No |
| 7 | KDM8 | lysine demethylase 8 [Source:HGNC Symbol;Acc:HGNC:25840] | 2594 | 0.291 | -0.0265 | No |
| 8 | TAF7 | TATA-box binding protein associated factor 7 [Source:HGNC Symbol;Acc:HGNC:11541] | 3035 | 0.246 | -0.0480 | No |
| 9 | MORC4 | MORC family CW-type zinc finger 4 [Source:HGNC Symbol;Acc:HGNC:23485] | 4525 | 0.111 | -0.1581 | No |
| 10 | NCAPD3 | non-SMC condensin II complex subunit D3 [Source:HGNC Symbol;Acc:HGNC:28952] | 4711 | 0.095 | -0.1676 | No |
| 11 | TP53BP1 | tumor protein p53 binding protein 1 [Source:HGNC Symbol;Acc:HGNC:11999] | 4946 | 0.076 | -0.1819 | No |
| 12 | CBX5 | chromobox 5 [Source:HGNC Symbol;Acc:HGNC:1555] | 4963 | 0.075 | -0.1792 | No |
| 13 | CDYL | chromodomain Y like [Source:HGNC Symbol;Acc:HGNC:1811] | 5016 | 0.072 | -0.1795 | No |
| 14 | ING1 | inhibitor of growth family member 1 [Source:HGNC Symbol;Acc:HGNC:6062] | 5146 | 0.060 | -0.1865 | No |
| 15 | PHF2 | PHD finger protein 2 [Source:HGNC Symbol;Acc:HGNC:8920] | 5332 | 0.045 | -0.1985 | No |
| 16 | MSL3 | MSL complex subunit 3 [Source:HGNC Symbol;Acc:HGNC:7370] | 5596 | 0.024 | -0.2178 | No |
| 17 | SPIN2A | spindlin family member 2A [Source:HGNC Symbol;Acc:HGNC:20694] | 5689 | 0.017 | -0.2240 | No |
| 18 | NCAPG2 | non-SMC condensin II complex subunit G2 [Source:HGNC Symbol;Acc:HGNC:21904] | 5700 | 0.016 | -0.2240 | No |
| 19 | MTF2 | metal response element binding transcription factor 2 [Source:HGNC Symbol;Acc:HGNC:29535] | 5996 | -0.004 | -0.2468 | No |
| 20 | ING2 | inhibitor of growth family member 2 [Source:HGNC Symbol;Acc:HGNC:6063] | 6150 | -0.016 | -0.2578 | No |
| 21 | THAP7 | THAP domain containing 7 [Source:HGNC Symbol;Acc:HGNC:23190] | 6412 | -0.036 | -0.2763 | No |
| 22 | ING3 | inhibitor of growth family member 3 [Source:HGNC Symbol;Acc:HGNC:14587] | 6518 | -0.043 | -0.2822 | No |
| 23 | L3MBTL1 | L3MBTL histone methyl-lysine binding protein 1 [Source:HGNC Symbol;Acc:HGNC:15905] | 6807 | -0.063 | -0.3013 | No |
| 24 | SGF29 | SAGA complex associated factor 29 [Source:HGNC Symbol;Acc:HGNC:25156] | 7363 | -0.107 | -0.3390 | No |
| 25 | MPHOSPH8 | M-phase phosphoprotein 8 [Source:HGNC Symbol;Acc:HGNC:29810] | 7413 | -0.112 | -0.3370 | No |
| 26 | PHF1 | PHD finger protein 1 [Source:HGNC Symbol;Acc:HGNC:8919] | 7429 | -0.113 | -0.3323 | No |
| 27 | RAG2 | recombination activating 2 [Source:HGNC Symbol;Acc:HGNC:9832] | 7462 | -0.115 | -0.3288 | No |
| 28 | ZZEF1 | zinc finger ZZ-type and EF-hand domain containing 1 [Source:HGNC Symbol;Acc:HGNC:29027] | 7490 | -0.118 | -0.3248 | No |
| 29 | ATRX | ATRX chromatin remodeler [Source:HGNC Symbol;Acc:HGNC:886] | 7551 | -0.123 | -0.3231 | No |
| 30 | FMR1 | FMRP translational regulator 1 [Source:HGNC Symbol;Acc:HGNC:3775] | 7944 | -0.151 | -0.3458 | No |
| 31 | CDY1 | chromodomain Y-linked 1 [Source:HGNC Symbol;Acc:HGNC:1809] | 8096 | -0.163 | -0.3490 | No |
| 32 | PWP1 | "PWP1 homolog, endonuclein [Source:HGNC Symbol;Acc:HGNC:17015]" | 8724 | -0.211 | -0.3869 | No |
| 33 | MSH6 | mutS homolog 6 [Source:HGNC Symbol;Acc:HGNC:7329] | 8846 | -0.221 | -0.3849 | No |
| 34 | ZCWPW1 | zinc finger CW-type and PWWP domain containing 1 [Source:HGNC Symbol;Acc:HGNC:23486] | 8865 | -0.222 | -0.3747 | No |
| 35 | ZMYND11 | zinc finger MYND-type containing 11 [Source:HGNC Symbol;Acc:HGNC:16966] | 8952 | -0.228 | -0.3696 | No |
| 36 | ZZZ3 | zinc finger ZZ-type containing 3 [Source:HGNC Symbol;Acc:HGNC:24523] | 8991 | -0.231 | -0.3605 | No |
| 37 | TDRD3 | tudor domain containing 3 [Source:HGNC Symbol;Acc:HGNC:20612] | 9080 | -0.238 | -0.3550 | No |
| 38 | PYGO1 | pygopus family PHD finger 1 [Source:HGNC Symbol;Acc:HGNC:30256] | 9628 | -0.286 | -0.3827 | No |
| 39 | TRIM24 | tripartite motif containing 24 [Source:HGNC Symbol;Acc:HGNC:11812] | 9927 | -0.314 | -0.3896 | Yes |
| 40 | MORC3 | MORC family CW-type zinc finger 3 [Source:HGNC Symbol;Acc:HGNC:23572] | 10037 | -0.325 | -0.3812 | Yes |
| 41 | CBX2 | chromobox 2 [Source:HGNC Symbol;Acc:HGNC:1552] | 10065 | -0.327 | -0.3663 | Yes |
| 42 | SUZ12 | SUZ12 polycomb repressive complex 2 subunit [Source:HGNC Symbol;Acc:HGNC:17101] | 10104 | -0.331 | -0.3520 | Yes |
| 43 | GLYR1 | glyoxylate reductase 1 homolog [Source:HGNC Symbol;Acc:HGNC:24434] | 10807 | -0.406 | -0.3855 | Yes |
| 44 | CXXC1 | CXXC finger protein 1 [Source:HGNC Symbol;Acc:HGNC:24343] | 10853 | -0.411 | -0.3677 | Yes |
| 45 | TTLL12 | tubulin tyrosine ligase like 12 [Source:HGNC Symbol;Acc:HGNC:28974] | 10861 | -0.411 | -0.3469 | Yes |
| 46 | KDM4A | lysine demethylase 4A [Source:HGNC Symbol;Acc:HGNC:22978] | 10936 | -0.423 | -0.3306 | Yes |
| 47 | KDM5A | lysine demethylase 5A [Source:HGNC Symbol;Acc:HGNC:9886] | 11097 | -0.443 | -0.3200 | Yes |
| 48 | CBX3 | chromobox 3 [Source:HGNC Symbol;Acc:HGNC:1553] | 11113 | -0.445 | -0.2981 | Yes |
| 49 | PHF8 | PHD finger protein 8 [Source:HGNC Symbol;Acc:HGNC:20672] | 11592 | -0.514 | -0.3086 | Yes |
| 50 | CHD5 | chromodomain helicase DNA binding protein 5 [Source:HGNC Symbol;Acc:HGNC:16816] | 11776 | -0.545 | -0.2945 | Yes |
| 51 | RBBP5 | "RB binding protein 5, histone lysine methyltransferase complex subunit [Source:HGNC Symbol;Acc:HGNC:9888]" | 11816 | -0.553 | -0.2687 | Yes |
| 52 | CHD8 | chromodomain helicase DNA binding protein 8 [Source:HGNC Symbol;Acc:HGNC:20153] | 11825 | -0.554 | -0.2406 | Yes |
| 53 | BPTF | bromodomain PHD finger transcription factor [Source:HGNC Symbol;Acc:HGNC:3581] | 11870 | -0.565 | -0.2146 | Yes |
| 54 | MBTD1 | mbt domain containing 1 [Source:HGNC Symbol;Acc:HGNC:19866] | 12189 | -0.641 | -0.2060 | Yes |
| 55 | ZMYND8 | zinc finger MYND-type containing 8 [Source:HGNC Symbol;Acc:HGNC:9397] | 12214 | -0.649 | -0.1741 | Yes |
| 56 | KDM7A | lysine demethylase 7A [Source:HGNC Symbol;Acc:HGNC:22224] | 12390 | -0.704 | -0.1512 | Yes |
| 57 | SPIN1 | spindlin 1 [Source:HGNC Symbol;Acc:HGNC:11243] | 12559 | -0.777 | -0.1238 | Yes |
| 58 | CBX8 | chromobox 8 [Source:HGNC Symbol;Acc:HGNC:15962] | 12586 | -0.787 | -0.0849 | Yes |
| 59 | CHD1 | chromodomain helicase DNA binding protein 1 [Source:HGNC Symbol;Acc:HGNC:1915] | 12745 | -0.908 | -0.0500 | Yes |
| 60 | TAF1 | TATA-box binding protein associated factor 1 [Source:HGNC Symbol;Acc:HGNC:11535] | 12881 | -1.192 | 0.0015 | Yes |
Table: GSEA details [plain text format]

  

Fig 2: GOMF\_METHYLATED\_HISTONE\_BINDING      
 Blue-Pink O' Gram in the Space of the Analyzed GeneSet

  

Fig 3: GOMF\_METHYLATED\_HISTONE\_BINDING: Random ES distribution      
 Gene set null distribution of ES for **GOMF\_METHYLATED\_HISTONE\_BINDING**
